# Arthroscopic Medial Meniscal Posterior Root Reconstruction Using Gracilis Autograft via the Anterior-Medial Collateral Ligament Portal to Prevent Meniscal Tissue Cut-Through

**DOI:** 10.1016/j.eats.2025.103611

**Published:** 2025-05-13

**Authors:** Nattha Kulkamthorn, Chonlapas Tungbutrawong, Theeraset Bantuchai, Tharit Inkaratana, Pattanaket Cheewakongkiat

**Affiliations:** a Sports Medicine Unit, Department of Orthopaedics, Phramongkutklao Hospital, Bangkok, Thailand; b Department of Orthopaedics, Ratchaburi Hospital, Ratchaburi, Thailand; c Department of Orthopaedics, Srisangworn Sukhothai Hospital, Sukhothai, Thailand

## Abstract

Meniscal root repair is the treatment of choice for reparable meniscal root tears; however, the complete healing rate is less than 60%. To improve healing outcomes, meniscal root reconstruction may serve as a viable alternative. We present an arthroscopic reconstructive technique using a gracilis graft through the anterior-medial collateral ligament portal. This approach offers the advantage of minimizing further meniscal tissue injury or cut-through.

Medial meniscal posterior root tears (MMPRTs) contribute to rapid progression of knee osteoarthritis^1^^,^^2^ due to biomechanical changes similar to total meniscectomy.^3^ Although meniscal root repair has been shown to improve clinical outcomes,^4^ healing rates remain suboptimal, with complete healing achieved in fewer than 60% of patients.^5^, ^6^, ^7^, ^8^ To address this, medial meniscal posterior root (MMPR) reconstruction using a gracilis graft has shown improved healing outcomes.^9^^,^^10^

Various techniques for MMPR reconstruction have been described in the literature,^11^, ^12^, ^13^, ^14^ each requiring different levels of technical expertise. A common challenge during these procedures is graft passage through the compromised tissue of a degenerative meniscus, which is prone to damage or cut-through. Approaches using the posteromedial portal have been proposed as potential solutions to this issue. This article describes a technique for MMPR reconstruction using a gracilis autograft via the anterior-medial collateral ligament (aMCL) portal. This approach aims to reduce the acute angle during tunnel dilation and optimize the direction of graft passage (Video 1).

## Surgical Technique

### Arthroscopic Confirmation of MMPRT and MMPR Footprint Preparation

Standard anterolateral (AL) and anteromedial (AM) portals are established. The MMPRT is confirmed by probing through the AM portal.

### Graft Harvesting and Preparation

A 4-cm vertical incision is made over the pes anserinus tendon, located 4 cm distal to the joint line and 2 cm medial to the tibial tuberosity. The sartorial sheath is incised 2 cm along the direction of its fibers, and the gracilis tendon is identified and harvested. A double-folded gracilis graft is prepared using No. 2 FiberWire (Arthrex, Naples, FL) via a whipstitch technique, resulting in a graft with a diameter of 6.5 mm and a length of 4 cm.

### Soft-Tissue Tunnel Creation

**Fig 1:**
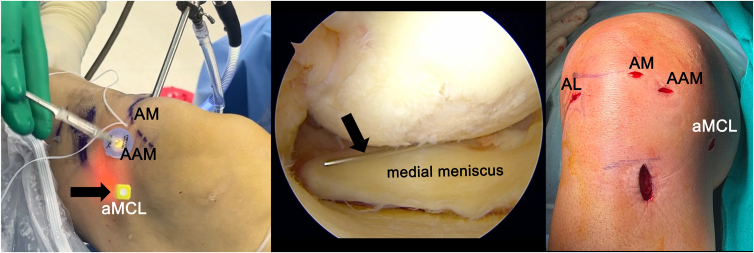
View from anterolateral portal (AL) in right knee. A 20-gauge spinal needle (arrows) is inserted into the joint. The entry point is identified at the intersection of the joint line and the medial epicondyle axis, ensuring placement near the upper surface of the meniscus. The anterior-medial collateral ligament portal (aMCL) is established with a vertical skin incision, parallel to the medial collateral ligament fibers to reduce the risk of medial collateral ligament injury. (AAM, accessory anteromedial portal; AM, anteromedial portal.)

**Fig 2:**
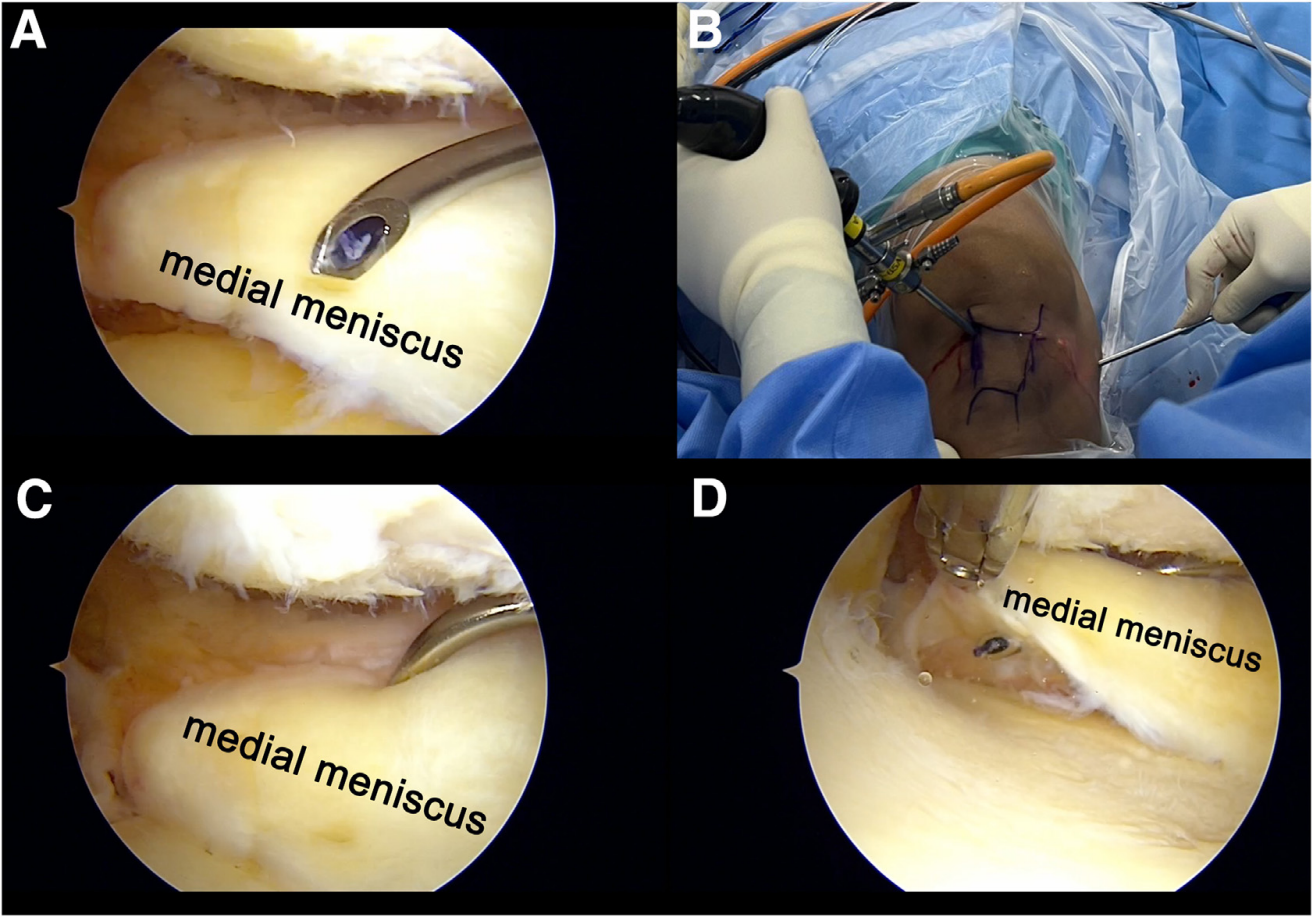
View from anterolateral portal in right knee. (A, B) To create the soft-tissue tunnel in the medial meniscus, an arthroscopic suture passer is inserted through the anterior-medial collateral ligament portal. (C, D) The suture lasso is used to pass through the meniscus, directing the suture from superior (C) to inferior (D). A No. 1 polydioxanone suture is passed for subsequent dilation of the meniscal tunnel using the multiple–mulberry knot suture.

To widen the joint space, the medial collateral ligament (MCL) is released using the pie-crusting technique. The aMCL portal is then created just anterior to the MCL at the level of the joint line, with MCL palpation and a light source used to transilluminate the skin for guidance (Fig 1). The site of the MMPRT is debrided and refreshed. Under visualization from the AL portal, a SutureLasso (Arthrex) is inserted through the aMCL portal, directed from superior to inferior, 1 cm medial to the tear edge and 1 mm anterior to the posterior edge of the meniscocapsular junction (Fig 2). A No. 1 polydioxanone suture (Ethicon) is passed from the aMCL portal and retrieved via the AM portal. This soft-tissue tunnel is then subsequently dilated to create the meniscal tunnel.

### Tibial Tunnel Preparation

**Fig 3:**
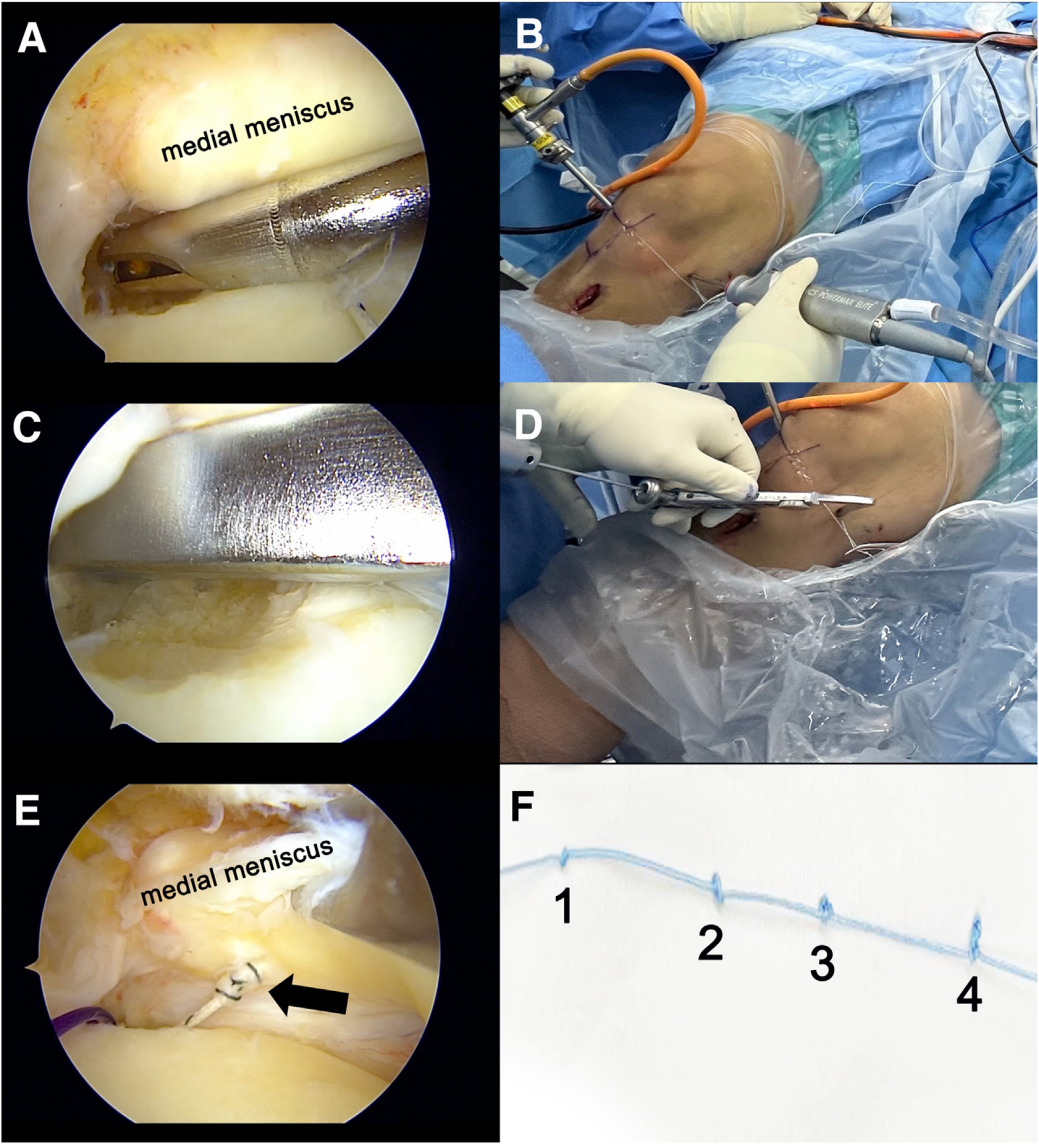
View from anterolateral portal in right knee. After preparation of the meniscal root footprint (A, B), the tibial tunnel is created (C, D). (E) The soft-tissue tunnel is dilated using the multiple–mulberry knot suture (arrow), which is passed from the anterior-medial collateral ligament portal through the tibial tunnel. (F) A multiple–mulberry knot suture is created using No. 2 high-strength braided suture, with the number indicating the total number of knots. It should be noted that the arthroscopic shaver and meniscal root guide are used through the anterior-medial collateral ligament portal, which provides a more favorable working angle.

While viewing from the AL portal, the surgeon prepares the MMPR footprint (Fig 3). A tibial tunnel is created using a tibial guide from the Meniscal Root System (Smith & Nephew, Memphis, TN) via the aMCL portal. After drilling of a 2.5-mm-diameter guide pin from the AM aspect of the proximal tibia through the meniscal root footprint and confirmation of the proper position, a 6.5-mm reamer is used to enlarge the tunnel (Fig 3C). A No. 1 polydioxanone loop is used for suture retrieval in a double-U loop fashion, facilitating the passage of multiple shuttle sutures through the tibial tunnel.

### Meniscal Soft-Tissue Tunnel Dilation

A multiple–mulberry knot suture is created using No. 2 high-strength braided suture (FiberWire), consisting of 4 sets of knots, with each set progressively increasing in the number of knots (first knot, 1 knot; second knot, 2 knots; third knot, 3 knots; and fourth knot, 4 knots) and spaced 1 cm apart (Fig 3F), to facilitate the sequential dilation of the soft-tissue tunnel. One limb of the multiple–mulberry knot suture is shuttled from the aMCL portal into the meniscal soft-tissue tunnel toward the tibial tunnel. The meniscal tunnel is then dilated using a seesaw technique.

### Graft Passage

**Fig 4:**
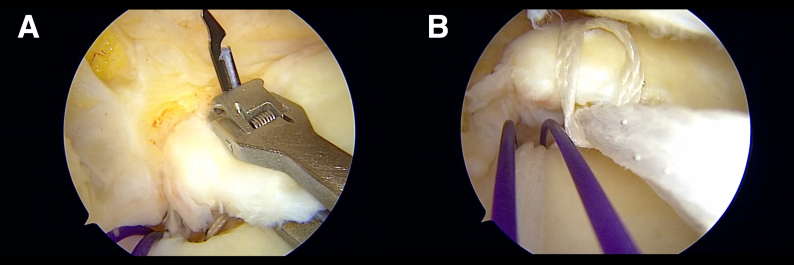
View from anterolateral (AL) portal in right knee. (A, B) The suture tape is shuttled to create a cinch stitch lateral to the previously established meniscal tunnel. A No. 1 polydioxanone loop (violet) is used for suture retrieval in a double-U loop fashion, enabling the passage of multiple shuttle sutures through the tibial tunnel.

**Fig 5:**
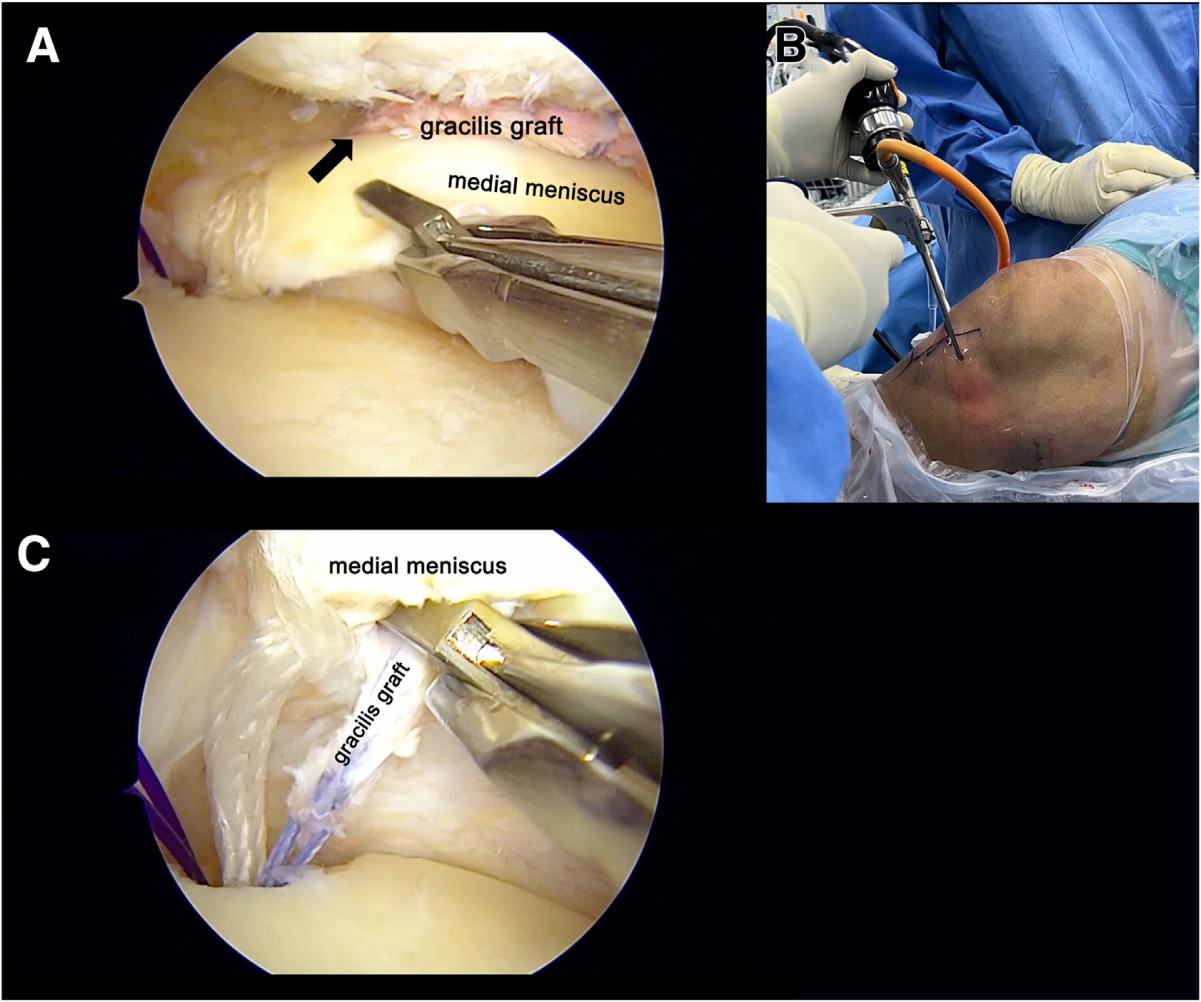
View from anterolateral (AL) portal in right knee. (A, B) The gracilis graft is passed through the anterior-medial collateral ligament portal and pulled through the meniscal tunnel (arrow) from superior to inferior. The meniscal tissue is stabilized during graft passage to prevent any potential injury to the meniscus. (C) The graft is passed from the inferior side of the meniscus into the tibial tunnel.

**Fig 6:**
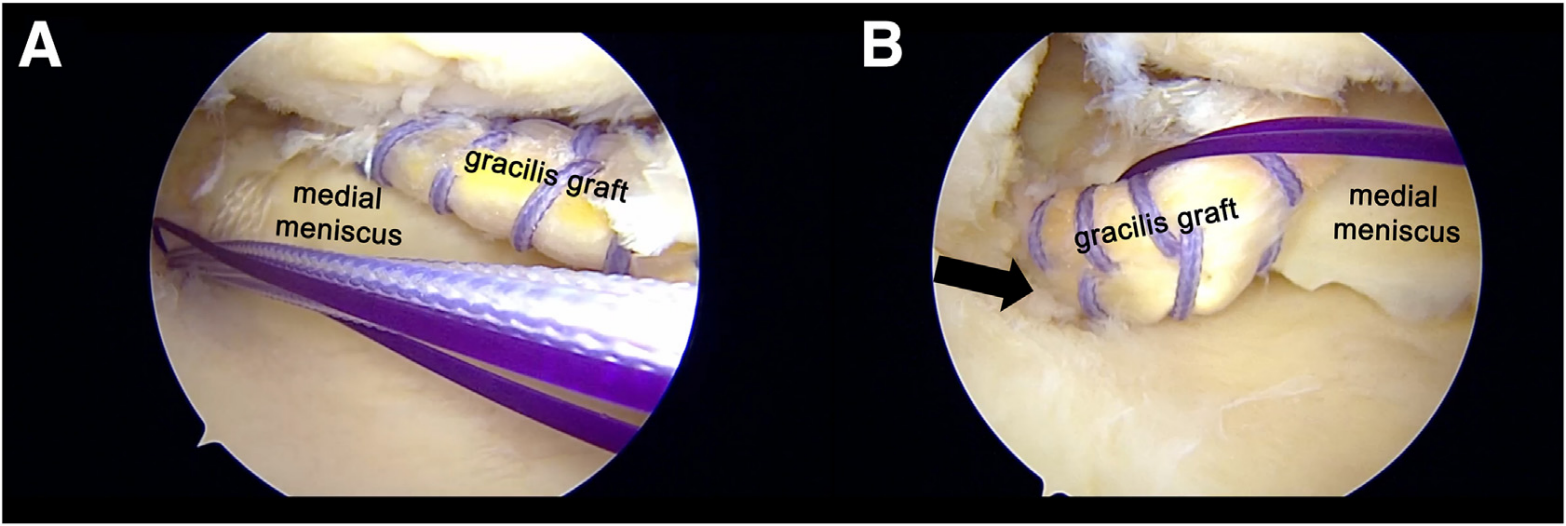
View from anterolateral (AL) portal in right knee. (A, B) The second arm of the graft is passed through the tibial tunnel (arrow) using double-U loop shuttle sutures.

A Knee Scorpion (Arthrex) is used to pass LabralTape (Arthrex) at 5 mm medial to the torn edge of the meniscal root, forming a cinch stitch (Fig 4). The suture tape is then shuttled into the tibial tunnel. The gracilis graft is passed through the meniscal tunnel from superior to inferior (Fig 5), with 1 arm of the graft passed through the tibial tunnel (Fig 6). The graft is advanced through the meniscus until its center is properly aligned with the meniscus, and the other end of the graft is then shuttled through the tibial tunnel.

### Fixation

**Fig 7:**
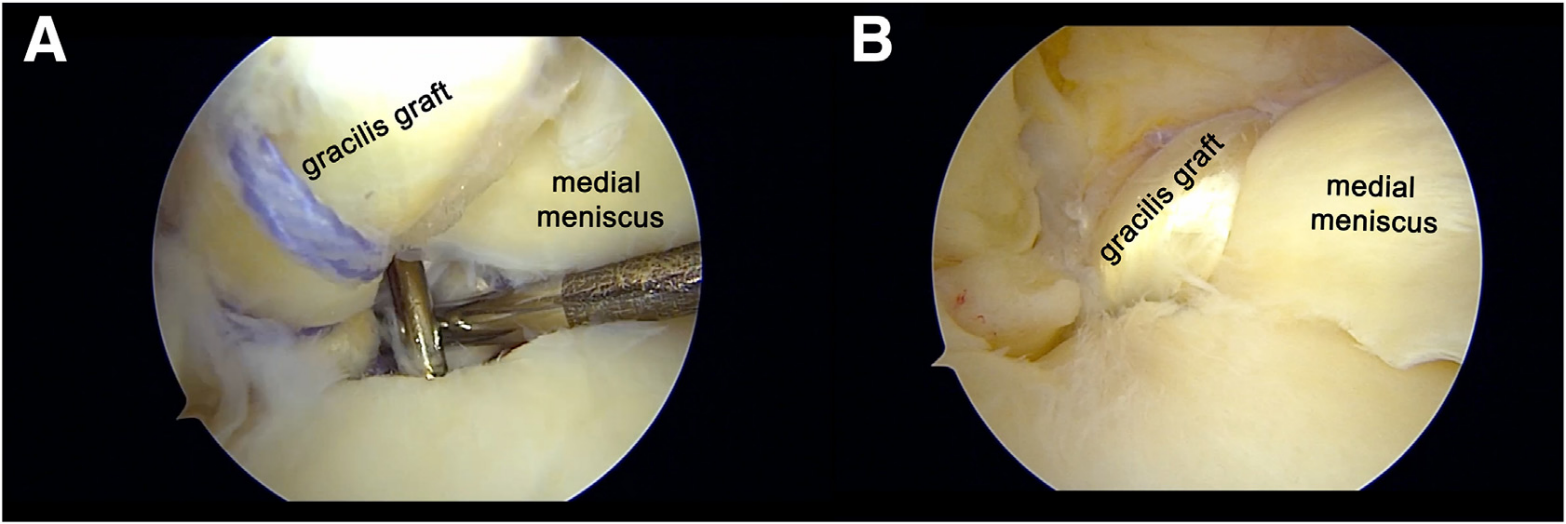
View from anterolateral (AL) portal in right knee. (A, B) A bioabsorbable interference screw is inserted while the graft is simultaneously tensioned under arthroscopic visualization.

A bioabsorbable interference screw (6 mm in diameter) is inserted into the tibial tunnel to secure the gracilis graft with aperture fixation while the sutures are simultaneously tensioned under arthroscopic visualization (Fig 7). The suture tape and remaining sutures are secured using an ABS button (Arthrex), with the knee positioned at approximately 30° of flexion. A final arthroscopic assessment is performed to confirm the integrity of the posterior root reconstruction and to evaluate the tension across the range of motion. Pearls and pitfalls are described in Table 1.

**Table 1:** Pearls and Pitfalls

Pearls
Sufficient working space should be established by performing an MCL release using the pie-crusting technique.
The meniscal soft-tissue tunnel should be sequentially dilated from the aMCL portal to the tibial tunnel.
The graft should be passed from the aMCL portal to the tibial tunnel in a superior-to-inferior direction.
The surgeon should use a double-U loop to shuttle the suture into the tibial tunnel, keeping it in place until both the graft and all sutures are completely passed.
Pitfalls
In cases of significant displacement or when the tear site is distant from the footprint, there is an increased risk of damaging the meniscus.

aMCL, anterior-medial collateral ligament; MCL, medial collateral ligament.

### Postoperative Rehabilitation

A few days after surgery, 50% weight-bearing walking in a full-extension knee brace is permitted, with closed-chain range of motion allowed with up to 90° of flexion. Patients are advised to bear weight as tolerated and can achieve full range of motion by 6 weeks postoperatively.

## Discussion

MMPRTs are associated with poor clinical outcomes if untreated.^1^^,^^2^ Krych et al.^1^ reported that 31% of patients with MMPRTs treated conservatively underwent total knee arthroplasty within 3 years. Although meniscal root repair can improve outcomes, there are compelling reasons to prefer meniscal root reconstruction: (1) the anatomy and histology of the meniscal root and (2) the low healing rates of meniscal root repair. Moreover, meniscal root reconstruction is indicated when little or no meniscal root tissue remains for fixation because repair may cause over-tensioning.^15^

Andrews et al.^16^ described the posterior meniscal root’s role in securely connecting the menisci to the tibial plateau, emphasizing its ligament-like structure integrating into the fibrocartilaginous composition of the meniscal body. Park et al.^17^ found that the histologic features of a healthy medial posterior meniscal root closely resemble those of ligaments and tendons, with fibrocartilage present only at the enthesis. Furthermore, Li et al.^18^ showed in a rabbit model that tendon grafts can replicate the structural properties of the meniscus, supporting the concept that tendon grafts aid in meniscal healing and facilitate fibrocartilaginous metaplasia. Thus, the gracilis graft may be a viable option for MMPR reconstruction.

The healing process associated with MMPR reconstruction using a gracilis graft addresses 3 critical interfaces: meniscus to graft, meniscus to bone, and bone to graft. This 3-point healing strategy enhances the likelihood of achieving complete MMPR healing and reduces the risk of complications from incomplete or failed healing.

Meniscal root repair is known for low healing rates. Seo et al.^6^ found that among 21 patients who underwent pull-out repair and returned for second-look arthroscopy, none achieved complete healing. Five showed lax healing, 4 exhibited scar healing, and 2 experienced failed healing. Feucht et al.^7^ reviewed studies on meniscal root repairs with a mean 30-month follow-up, discovering that meniscal extrusion was reduced in 56% of cases, whereas complete healing—evaluated through magnetic resonance imaging or second-look arthroscopy—was found in 62% of cases. Additionally, Jung et al.^5^ studied 13 patients with MMPRTs who underwent all-inside repair using a suture anchor, resulting in only a 50% healing rate per magnetic resonance imaging. Li et al.^9^ found an improved healing rate of 82% for meniscal root reconstruction, in contrast to 51% for meniscal root repair.

Nevertheless, MMPR reconstruction still requires significant technical skill. In chronic cases, a challenge is the poor tissue quality of the remaining stump and degenerative meniscus, increasing the risk of damaging or cutting through the meniscal tissue during graft passage.^13^ Our technique uses the aMCL portal, which runs parallel to the MMPR fibers, providing easier access to the reconstruction site during procedures such as suture passing, guide drilling, and meniscal tunnel dilation. This approach also helps reduce the “killer turn,” facilitating easier graft passage from the aMCL portal to the tibial tunnel. Additionally, the aMCL portal offers greater working space, simplifying the dilation of the meniscal soft-tissue tunnel. During passage of the graft from the aMCL to the tibial tunnel, visualization occurs through the AL portal, whereas the AM portal stabilizes the meniscal tissue during knot tying or graft passage.

In summary, we present the aMCL portal technique for MMPR reconstruction using gracilis autograft. Our technique provides several advantages that may simplify the surgical procedure, warranting further studies to assess clinical outcomes and healing rates associated with this approach. The advantages and disadvantages of this technique are outlined in Table 2.

**Table 2:** Advantages and Disadvantages

Advantages
The necessity for a posterior portal is eliminated.
There is increased availability of portals for surgical manipulation.
Reconstruction of the medial meniscal root using an autologous tendon graft can restore meniscal hoop stress function while preserving the physiological excursion of the meniscal root.
Pull-out repair increases initial repair strength and reduces graft stress during the early stages.
Disadvantages
The technique is more complex and demands a higher level of surgical skill than simple repairs.
There is potential for donor-site morbidity associated with harvesting the gracilis graft.
Graft passage would be difficult in cases of poor-quality menisci.

## Disclosures

All authors (N.K., C.T., T.B., T.I., P.C.) declare that they have no known competing financial interests or personal relationships that could have appeared to influence the work reported in this paper.

## Supplementary Data

Video 1 An example is presented to demonstrate the technique and advantages of medial meniscal root reconstruction. The procedure is performed on a right knee in the supine position with a thigh tourniquet. Preferred tunnel placement, tibial footprint preparation, and suture passage techniques are shown. The anterior-medial collateral ligament (aMCL) portal is used for improved suture passage. This approach aims to reduce the acute angle during tunnel dilation and optimize the direction of graft passage. Sutures are passed through the meniscal root and pulled through the tibial tunnel for secure fixation. This technique can restore meniscal hoop stress function while preserving the physiological excursion of the meniscal root, enhancing stability and load distribution.
